# Temporal Increase in Strict Spontaneous Intracerebral Hemorrhage Admissions During the First March Following Direct Israel–Iran Hostilities: Preliminary Single-Center Findings from a Decade-Referenced Neuroscience Services Cohort

**DOI:** 10.3390/ijerph23060772

**Published:** 2026-06-08

**Authors:** Paz Kelmer, Shachar Zion Shemesh, Jose Asprilla, Omri Cohen, Zvi R. Cohen, Lior Ungar

**Affiliations:** 1 Department of Neurosurgery, Sheba Medical Center, Ramat Gan 6997801, Israel; pazkelmer92@gmail.com (P.K.); asprilla_05@hotmail.com (J.A.);; 2 Gray Faculty of Medicine, Tel Aviv University, Tel Aviv 6997801, Israel; 3 Dina Recanati School of Medicine, Reichman University, Herzeliya 4610101, Israel; 4 Pain Center, Sheba Medical Center, Ramat Gan 6997801, Israel

**Keywords:** intracerebral hemorrhage, spontaneous ICH, war, wartime stress, blood pressure, psychological stress, seasonality, hemorrhagic stroke, neurosurgery, conflict, cardiovascular risk

## Abstract

**Highlights:**

**Public health relevance—How does this work relate to a public health issue?**

- This study addresses the indirect health effects of armed conflict by examining whether the early wartime period was temporally associated with an increased proportional burden of strict spontaneous intracerebral hemorrhage admissions within a single-center neurology/neurosurgery services dataset.
- It links conflict-related stressors, including repeated alerts, sleep disruption, healthcare reorganization, and possible medication nonadherence, to a major cerebrovascular outcome with substantial morbidity, mortality, and healthcare burden.

**Public health significance—Why is this work of significance to public health?**

- During March 2026, strict spontaneous intracerebral hemorrhage accounted for 4.0% of admissions versus 1.6% across March cohorts from the preceding decade, corresponding to a rate ratio of 2.48, while acute ischemic stroke did not increase. This suggests a more specific service-level increase in strict spontaneous intracerebral hemorrhage admissions rather than a general rise in cerebrovascular admissions.
- The observed excess was concentrated largely in older patients, highlighting the vulnerability of high-risk populations and reinforcing the public health importance of preserving chronic disease control during periods of armed conflict.

**Public health implications—What are the key implications or messages for practitioners, policymakers, and/or researchers in public health?**

- Practitioners and health systems should intensify wartime blood pressure surveillance, support antihypertensive adherence, and incorporate psychological and behavioral support for patients with vascular risk factors, particularly older adults.
- Policymakers and researchers should recognize armed conflict as a driver of indirect cerebrovascular harm and prioritize multicenter and registry-based studies to confirm these findings and guide conflict-sensitive prevention strategies.

**Abstract:**

**Objective**: On 28 February 2026, Israel entered direct large-scale hostilities with Iran under Operation Roaring Lion. The opening phase was characterized by repeated missile alerts, civilian protected-space instructions, and rapid reorganization of hospital activity into protected areas. We observed an apparent increase in strict spontaneous intracerebral hemorrhage admissions during March 2026 within our linked neurology/neurosurgery services dataset. The aim of this preliminary single-center study was to determine whether March 2026 was temporally associated with a higher proportional burden of strict spontaneous intracerebral hemorrhage admissions compared with March cohorts from the preceding decade and whether this pattern was also observed for acute ischemic stroke or non-traumatic subarachnoid hemorrhage. **Methods**: We performed a retrospective observational cohort study of all unique March admissions captured within a linked neurology/neurosurgery services dataset from 2016 through 2026. Hospitalizations were deduplicated by admission number. March 2026 was treated as the first full March occurring after the onset of direct Israel–Iran hostilities on 28 February 2026. Strict spontaneous ICH was defined using diagnosis-text phenotyping that included intraparenchymal or intracerebral hemorrhage terminology while excluding trauma, subarachnoid hemorrhage, subdural hematoma, aneurysm, arteriovenous malformation, tumor-related hemorrhage, cavernoma, venous sinus thrombosis, dissection, and other clearly secondary etiologies. Comparator phenotypes included acute ischemic stroke and non-traumatic subarachnoid hemorrhage (SAH). **Results**: Across 3855 unique March admissions, 68 met criteria for strict spontaneous ICH. In March 2026, 9 of 223 admissions (4.0%) were classified as strict spontaneous ICH, compared with 59 of 3632 admissions (1.6%) across March 2016–2025, yielding a rate ratio of 2.48 (95% CI 1.25–4.94; *p* = 0.015). Patients with strict spontaneous ICH in March 2026 were older (mean age 72.3 vs. 65.8 years), and 7 of 9 cases (77.8%) occurred in patients aged ≥70 years compared with 25 of 59 (42.4%) historically (*p* = 0.073). Acute ischemic stroke did not increase in March 2026 (7.6% vs. 9.4%; *p* = 0.475), and non-traumatic SAH showed only a non-significant numerical increase (2.7% vs. 1.4%; *p* = 0.147). Sensitivity analyses showed a directionally consistent but statistically non-significant increase when March 2026 was compared with March 2025 alone (4.0% vs. 1.2%; rate ratio 3.36, 95% CI 0.92–12.27; *p* = 0.076) and with a rolling 3-year March baseline from 2023 through 2025 (4.0% vs. 2.1%; rate ratio 1.93, 95% CI 0.88–4.23; *p* = 0.143). In-hospital mortality among strict spontaneous ICH patients was 1 of 9 (11.1%) in March 2026 versus 4 of 59 (6.8%) in March 2016–2025. **Conclusions**: In this preliminary single-center neurology/neurosurgery services cohort, March 2026 showed a higher proportional burden of strict spontaneous intracerebral hemorrhage admissions than March cohorts from the preceding decade, while acute ischemic stroke did not increase. Sensitivity analyses using March 2025 alone and a rolling 3-year March baseline were directionally consistent but did not reach statistical significance. These findings should therefore be interpreted as a hypothesis-generating temporal association rather than evidence of causality or population-level incidence. Wartime-related psychological stress, sleep disruption, altered healthcare access, blood pressure dysregulation, and medication nonadherence are biologically plausible contributors, but individual-level blood pressure, medication exposure, body mass index, time-to-admission, direct stress exposure, and detailed outcome data were not available in the present dataset. Multicenter, hospital-wide, and registry-based validation with seasonal and systems-level sensitivity analyses is required.

## 1. Introduction

On 28 February 2026, direct large-scale Israel–Iran hostilities began under Operation Roaring Lion. Official Israel Defense Forces communications described the launch of a broad military operation, while Home Front Command instructions repeatedly directed civilians to remain in protected spaces during missile alerts and barrages [1,2]. This period created an unusual civilian and hospital-operational context in which acute neurological admissions required continued management under wartime constraints [3,4].

Spontaneous intracerebral hemorrhage (ICH) is among the most devastating forms of stroke, carrying high early mortality, severe neurological disability, prolonged hospitalization, and substantial neurocritical care utilization [5,6]. Contemporary reviews and guideline statements continue to identify spontaneous ICH as a major neurological emergency with a persistent global burden despite advances in stroke systems, neuroimaging, and critical care [5,6,7]. The clinical importance of ICH extends beyond individual patient outcome: even small fluctuations in admission burden can materially affect neurosurgical consultation volume, ICU occupancy, acute transfer pathways, and the need for urgent invasive monitoring or operative intervention.

Beyond its established vascular risk factors, spontaneous ICH exhibits meaningful temporal patterning. Population-scale studies increasingly suggest that ICH occurrence is not randomly distributed across the calendar year. Large datasets from the United States and Germany have shown that the incidence of primary ICH rises during colder periods, and a regional Israeli study similarly demonstrated seasonal variation in spontaneous ICH [8,9]. A 21-year Israeli population-based study found that the seasonal signal of stroke, including ICH, strengthened with advancing age, with hemorrhagic events showing a winter/autumn predominance [10]. These observations support the concept that older patients may be especially vulnerable to short-term environmental or physiological triggers superimposed on chronic vascular fragility [11,12,13,14,15].

Several mechanisms could plausibly amplify hemorrhagic cerebrovascular risk during wartime conditions. Acute psychological stress has been associated with an increased risk of stroke in meta-analytic data, and such stress may be particularly relevant during periods characterized by repeated sirens, disrupted sleep, displacement into protected spaces, abrupt interruption of routine, and sustained uncertainty [14]. At the physiological level, these exposures may plausibly contribute to sympathetic nervous system activation, transient blood pressure elevation, medication nonadherence, dehydration, and loss of chronic disease stability. Each of which could increase susceptibility to spontaneous intracranial bleeding in vulnerable patients [14,15,16,17]. Although the current dataset does not allow direct measurement of these intermediate variables, the wartime setting provides a biologically coherent context in which a service-level temporal increase in strict spontaneous intracerebral hemorrhage admissions could plausibly occur [14,16,17].

Environmental cofactors may further interact with wartime physiology. Prior studies have linked spontaneous ICH occurrence to low ambient temperature, humidity-related effects, and selected air pollutants, and newer case-crossover work suggests that extreme temperature and low humidity may act as short-latency triggers of acute ICH, particularly in older adults [12,13]. By contrast, the literature on subarachnoid hemorrhage (SAH) seasonality is more heterogeneous: some cohorts and meta-analytic syntheses suggest modest seasonal or meteorological effects, whereas others show weaker or inconsistent patterns [15,16]. This distinction is important because it creates an opportunity to examine whether any March 2026 wartime excess was broadly hemorrhagic or more specifically concentrated in spontaneous intraparenchymal hemorrhage.

Wartime conditions may also alter the hospital-facing phenotype of cerebrovascular disease independent of biology. Changes in referral pathways, prehospital transport behavior, thresholds for presentation, family decision-making, and service reorganization may all influence which patients ultimately appear within neurology and neurosurgery datasets. For this reason, any apparent spike in hemorrhagic admissions during wartime must be interpreted cautiously: it may represent a true change in event frequency, a change in case capture, or a combination of both. A structured comparison against a stable historical reference frame is therefore essential before stronger mechanistic or causal interpretations are entertained.

During March 2026, we observed an apparent increase in strict spontaneous intracerebral hemorrhage admissions within our neurology/neurosurgery services dataset. We therefore performed a preliminary, single-center, decade-referenced analysis comparing March 2026 with March cohorts from 2016 through 2025. The primary aim was to determine whether March 2026 was temporally associated with an increased proportional burden of strict spontaneous intracerebral hemorrhage admissions within the same services dataset. Secondary aims were to evaluate whether any observed increase was also seen for acute ischemic stroke or non-traumatic subarachnoid hemorrhage and to frame the findings cautiously in relation to seasonality, hospital case capture and wartime changes in referral and admission patterns.

## 2. Methods

### 2.1. Study Design and Data Sources

We performed a retrospective observational cohort study using two linked spreadsheet-based datasets derived from admissions captured within neurology and neurosurgery services at Sheba Medical Center. The primary file contained admission number, department, admission timing, diagnosis text, and related hospitalization variables. A secondary file contained demographic variables, including date of birth and sex. Records were linked using the admission number, allowing row-level merge integrity.

The dataset should therefore be interpreted as a neurology/neurosurgery services dataset rather than a hospital-wide stroke registry or population-based epidemiological dataset.

### 2.2. Ethics Approval

The study was conducted in accordance with the Declaration of Helsinki and was approved by the Sheba Medical Center Institutional Review Board (protocol code 8148-21-SMC; initial approval date: 26 January 2021; renewal approval date: 9 December 2025; valid until 7 March 2027). The requirement for informed consent was waived because of the retrospective design and the use of anonymized registry-based clinical data.

### 2.3. Wartime Exposure Window

The analytic frame was restricted to March admissions from 2016 through 2026. March 2026 was defined a priori as the first full March cohort occurring after the onset of direct Israel–Iran hostilities on 28 February 2026. Historical comparator cohorts consisted of March admissions from 2016 through 2025.

### 2.4. Hospital Operating Context During the Wartime Period

During March 2026, hospital activity occurred under wartime constraints, including protected-space alerts, changes in hospital operating routines, and reorganization of selected clinical activity into protected areas. These conditions may have influenced emergency transport behavior, interhospital transfer patterns, thresholds for admission, diversion from surrounding facilities, and the relative distribution of cases captured within neurology and neurosurgery services. Because the dataset was service-based, the analysis cannot distinguish a true change in regional intracerebral hemorrhage incidence from changes in case capture, referral pathways, or hospital-facing clinical burden.

### 2.5. Deduplication and Unit of Analysis

Because departmental datasets may include repeated entries related to the same hospitalization, all records were deduplicated at the hospitalization level using the admission number. The final analytic dataset contained 3855 unique March admissions.

### 2.6. Primary Case Definition

The primary phenotype was strict spontaneous ICH. Cases were identified through diagnosis-text review. Inclusion terms captured intracerebral hemorrhage, cerebral hemorrhage, parenchymal hemorrhage, cerebellar hemorrhage, brainstem or pontine hemorrhage, and generic intracranial hemorrhage when the broader text context was compatible with spontaneous intraparenchymal hemorrhage. Exclusion criteria included explicit evidence of trauma, subarachnoid hemorrhage, subdural hematoma, epidural hematoma, aneurysm, arteriovenous malformation, tumor-related hemorrhage, cavernoma, venous sinus thrombosis, hemorrhagic transformation of ischemic stroke, dissection, or another clearly secondary cause.

### 2.7. Comparator Phenotypes

Two comparator phenotypes were examined. Acute ischemic stroke was defined by diagnosis text consistent with acute cerebral infarction or ischemic stroke, excluding transient ischemic attack and hemorrhagic stroke phenotypes. Non-traumatic SAH was defined by diagnosis text indicating subarachnoid hemorrhage without evidence of trauma.

### 2.8. Covariates and Subtypes

Age at admission was calculated from date of birth. Sex was extracted directly from the demographic dataset. Within the strict spontaneous ICH cohort, hemorrhage subtype was secondarily categorized as parenchymal cerebral ICH, ICH with intraventricular extension, unspecified or generic intracranial hemorrhage, cerebellar ICH, or brainstem/pontine ICH.

### 2.9. Statistical Analysis

The principal comparison was between observed March 2026 admissions and pooled March 2016–2025 admissions. For rate-based analyses, denominators were total admissions captured in the same neurology/neurosurgery services dataset: 223 admissions in March 2026 and 3632 admissions across March 2016–2025. Continuous variables were compared using the Mann–Whitney U test, categorical variables using Fisher’s exact test, and effect size was summarized as rate ratio with a 95% confidence interval. Analyses were performed using Python 3.13, including pandas for data handling and scipy.stats for statistical testing.

To evaluate robustness, sensitivity analyses compared March 2026 with March 2025 alone and with a rolling 3-year March baseline from 2023 through 2025. Two-sided Fisher exact tests were used for all proportional comparisons, and effect sizes were summarized as rate ratios with 95% confidence intervals. This study should be interpreted as a preliminary single-center services-level analysis rather than a hospital-wide incidence study.

Because the available linked dataset was structured for March-matched comparison, formal all-month seasonal modeling was not performed. The March-to-March design partially reduces seasonal confounding by comparing the same calendar month across years, but it does not eliminate the possibility of residual seasonal, secular, or systems-level confounding.

## 3. Results

### 3.1. Overall Cohort

Across the 11 March cohorts from 2016 through 2026, the linked dataset contained 3855 unique admissions, of which 68 met the strict spontaneous ICH phenotype. The source dataset was dominated by non-ICH neurological or neurosurgical hospitalizations, making strict spontaneous ICH a relatively infrequent but clinically important event class.

### 3.2. Primary Wartime March Comparison

In March 2026, 9 of 223 admissions (4.0%) were classified as strict spontaneous ICH. Across March 2016–2025, 59 of 3632 admissions (1.6%) met the same definition. This corresponded to a rate ratio of 2.48 (95% CI 1.25–4.94; Fisher *p* = 0.015), indicating that the proportional burden of strict spontaneous ICH during March 2026 was approximately two and a half times the historical March baseline within the same services dataset.

### 3.3. Age Profile of the ICH Cohort

**Figure 1:**
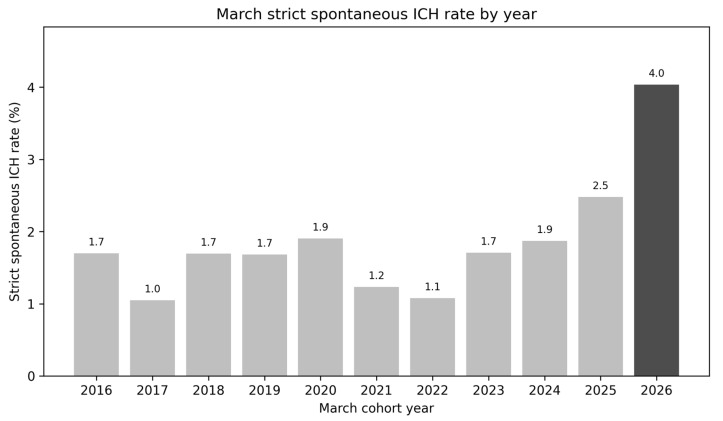
Year-by-year proportion of March admissions classified as strict spontaneous intracerebral hemorrhage within the neurology/neurosurgery services dataset.

**Figure 2:**
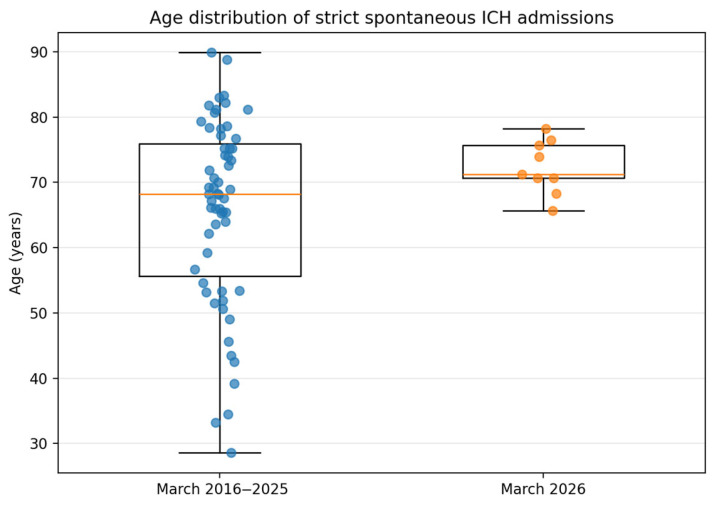
Age distribution of strict spontaneous intracerebral hemorrhage cases in March 2026 versus the pooled March 2016–2025 cohort.

**Figure 3:**
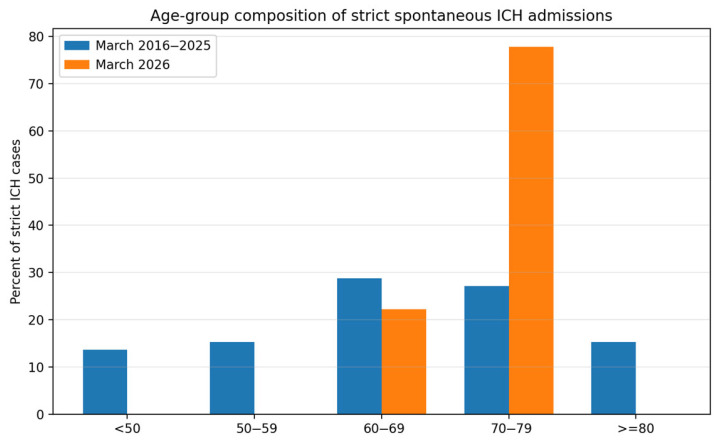
Age-group distribution of strict spontaneous intracerebral hemorrhage cases, demonstrating concentration of March 2026 cases in the 70–79-year age band.

Patients in the March 2026 strict spontaneous ICH cohort were older overall than those in the pooled historical March cohort. For March 2026, the mean age was 72.3 years, the median age was 71.2 years, and the interquartile range was 70.7–75.7 years. For March 2016–2025, the mean age was 65.8 years, the median age was 68.2 years, and the interquartile range was 55.7–76.0 years. Although the overall age comparison did not reach statistical significance (Mann–Whitney *p* = 0.215), 7 of 9 March 2026 ICH cases (77.8%) occurred in patients aged 70 years or older, compared with 25 of 59 historical cases (42.4%) (Fisher *p* = 0.073). The March 2026 ICH cohort was concentrated in the 70–79-year age band (Table 1; Figure 1, Figure 2 and Figure 3).

### 3.4. Sex Distribution

Sex composition was similar across cohorts. In March 2026, 6 of 9 patients (66.7%) were male and 3 of 9 (33.3%) were female. In the historical cohort, 43 of 59 (72.9%) were male and 16 of 59 (27.1%) were female (Fisher *p* = 0.702).

### 3.5. Hemorrhage Subtype Composition

**Figure 4:**
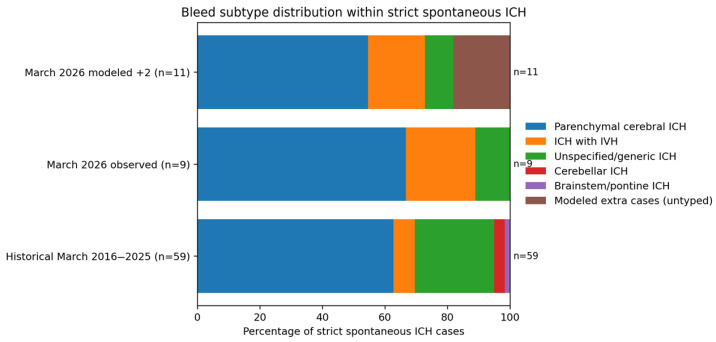
Hemorrhage subtype distribution in March 2026 versus March 2016–2025, including parenchymal cerebral ICH, ICH with intraventricular extension, and other strict spontaneous ICH categories.

The subtype profile of March 2026 strict spontaneous ICH remained broadly similar to that of prior March cohorts. In March 2026, 6 of 9 cases (66.7%) were parenchymal cerebral ICH, 2 of 9 (22.2%) were ICH with intraventricular extension, and 1 of 9 (11.1%) was unspecified or generic intracranial hemorrhage. In March 2016–2025, corresponding values were 37 of 59 (62.7%), 4 of 59 (6.8%), and 15 of 59 (25.4%), with 2 cerebellar hemorrhages and 1 brainstem/pontine hemorrhage also present historically. The numerical increase in intraventricular hemorrhage-associated presentations in March 2026 did not reach statistical significance (Fisher *p* = 0.177) (Figure 1 and Figure 4).

### 3.6. Comparator Cerebrovascular Phenotypes

**Figure 5:**
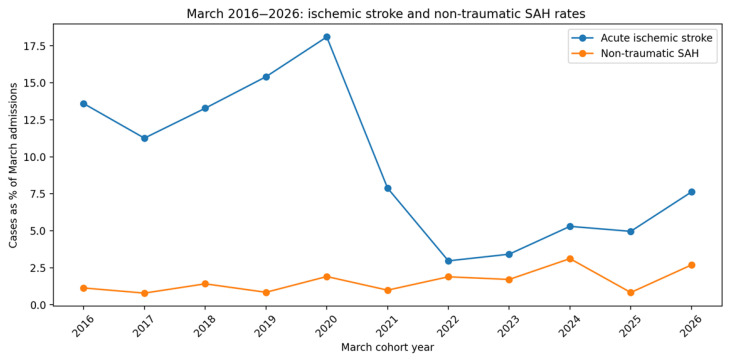
Compare phenotype rates in March 2026 versus pooled March 2016–2025 for strict spontaneous ICH, acute ischemic stroke, and non-traumatic subarachnoid hemorrhage.

Comparator phenotype analysis did not demonstrate a generalized rise across all cerebrovascular admissions. Acute ischemic stroke was present in 17 of 223 March 2026 admissions (7.6%), compared with 342 of 3632 historical March admissions (9.4%), yielding no evidence of increase (rate ratio 0.81, 95% CI 0.51–1.29; Fisher *p* = 0.475). The March 2026 ischemic stroke cohort was significantly older than the historical comparator cohort, with a mean age of 78.1 versus 67.9 years (Mann–Whitney *p* = 0.0012). Fourteen of 17 March 2026 ischemic stroke cases (82.4%) occurred in patients aged 70 years or older, compared with 158 of 342 historical cases (46.2%) (Fisher *p* = 0.0049). Non-traumatic SAH showed only a numerical, non-significant increase: 6 of 223 March 2026 admissions (2.7%) versus 52 of 3632 historical March admissions (1.4%), corresponding to a rate ratio of 1.88 (95% CI 0.82–4.33; Fisher *p* = 0.147). Age and sex distributions were similar between the March 2026 and historical SAH cohorts (Table 2; Figure 5).

### 3.7. Sensitivity Analyses

Sensitivity analyses were performed to evaluate whether the primary March 2026 finding was robust to alternative historical reference periods. Compared with March 2025 alone, spontaneous strict intracerebral hemorrhage accounted for 9 of 223 admissions in March 2026 (4.0%) versus 3 of 250 admissions in March 2025 (1.2%), corresponding to a rate ratio of 3.36 (95% CI 0.92–12.27; Fisher exact *p* = 0.076).

Using a rolling 3-year March baseline from 2023 through 2025, strict spontaneous intracerebral hemorrhage accounted for 18 of 860 admissions (2.1%), compared with 9 of 223 admissions in March 2026 (4.0%). This yielded a rate ratio of 1.93 (95% CI 0.88–4.23; Fisher exact *p* = 0.143).

Year-specific comparisons showed the same direction of effect but did not reach statistical significance. March 2026 was higher than March 2023 (9/223 [4.0%] vs. 7/285 [2.5%]; rate ratio 1.64, 95% CI 0.62–4.34; *p* = 0.320) and March 2024 (9/223 [4.0%] vs. 8/325 [2.5%]; rate ratio 1.64, 95% CI 0.64–4.18; *p* = 0.323). Overall, these sensitivity analyses were directionally consistent with the primary decade-referenced finding but were underpowered and did not independently confirm statistical robustness.

## 4. Discussion

The principal finding of this preliminary single-center study is that March 2026 was temporally associated with a higher proportional burden of strict spontaneous intracerebral hemorrhage admissions within our linked neurology/neurosurgery services dataset. Strict spontaneous intracerebral hemorrhage accounted for 9 of 223 March 2026 admissions (4.0%) compared with 59 of 3632 March admissions from 2016 to 2025 (1.6%). This finding should be interpreted as a services-level temporal association rather than evidence of causality or population-level incidence. Importantly, the same pattern was not observed for acute ischemic stroke, and non-traumatic subarachnoid hemorrhage showed only a non-significant numerical increase, suggesting that the finding was not simply a uniform increase across all cerebrovascular admissions.

This observation must also be interpreted within the broader literature on seasonal ICH epidemiology. Large-scale analyses from the United States, northern Israel, and broader Israeli population data have shown that spontaneous ICH incidence varies by season and tends to rise during colder periods [8,9,10]. The Israeli age-stratified population study is especially relevant because it demonstrated that the seasonal signal strengthens with increasing age [10]. The March-matched design of the present study partially addresses seasonal confounding by comparing March 2026 with March cohorts from prior years, but it cannot fully substitute for all-month seasonal modeling. In the current dataset, the March 2026 ICH cohort trended older and clustered strongly in the 70–79-year age band. Although the age comparison did not cross the conventional threshold for statistical significance, the direction of effect was coherent and clinically meaningful.

The wartime context provides additional and compelling biological plausibility without establishing causality. The opening phase of the conflict was characterized by repeated missile alerts, widespread civilian disruption, and major healthcare reorganization, including underground relocation of clinical activity [1,2,3,4]. Psychological stress has been associated with increased stroke risk in meta-analyses [14], and studies of temperature and air pollution have linked selected environmental exposures with increased ICH occurrence [12,13]. In practical terms, acute stress, sleep disruption, missed medications, blood pressure instability, dehydration, delayed presentation, and systems-level rerouting may all have contributed to the observed increase, although the present dataset cannot directly test those mechanisms.

### Clinical and Systems-Level Interpretation

The observed temporal increase is biologically plausible but cannot be mechanistically proven from the present dataset. Wartime conditions may increase psychological stress, sleep disruption, sympathetic activation, blood pressure instability, dehydration, and medication nonadherence, all of which are relevant to spontaneous intracerebral hemorrhage risk in older patients with chronic vascular disease [5,6,14,17,18,19,20,21,22,23,24,25]. However, the present dataset did not include presenting blood pressure, antihypertensive adherence, anticoagulant exposure, body mass index, direct stress measures, time-to-admission, hematoma volume, or detailed functional outcome data, and therefore cannot determine which of these mechanisms, if any, contributed to the observed pattern [18,19,20,21].

The clinical implication is not that wartime exposure causes intracerebral hemorrhage, but that patients with vascular risk factors may require more active chronic disease support during periods of major civilian disruption. In practical terms, primary care physicians, internists, neurologists, and emergency systems may consider reinforcing blood pressure monitoring, medication continuity, telemedicine follow-up, and early evaluation of neurological symptoms among older or hypertensive patients during wartime.

At the same time, several systems-level explanations must be considered. Wartime may alter emergency transport behavior, interhospital transfers, neurosurgical triage thresholds, diversion from surrounding hospitals, family decisions to seek care, and the probability that a case is captured within a tertiary neurology/neurosurgery services dataset. These factors are especially important because the denominator in this study reflects service-level admissions rather than a fixed population catchment. The results therefore indicate a hospital-facing increase in strict spontaneous intracerebral hemorrhage burden within this dataset, not a confirmed increase in regional incidence.

From a neurosurgical perspective, even a preliminary service-level increase in strict spontaneous intracerebral hemorrhage admissions may be relevant. Spontaneous intracerebral hemorrhage is resource-intensive, frequently requires urgent neurological, neurosurgical, and neurocritical care evaluation, and may affect ICU occupancy, imaging pathways, transfer decisions, and operative readiness. Therefore, the present findings are best understood as an early signal relevant to neuroscience service preparedness during wartime, rather than as definitive epidemiological evidence.

## 5. Limitations

This study has several important limitations. First, it is a retrospective, single-center, service-level analysis based on neurology/neurosurgery admissions rather than a hospital-wide stroke registry or a population-based epidemiological dataset. The findings therefore cannot be generalized to regional incidence. Second, the March 2026 strict spontaneous intracerebral hemorrhage cohort was small, with only 9 cases, and the results should be interpreted as preliminary and hypothesis-generating. Third, the dataset lacked several clinically important variables requested for more detailed characterization, including height, weight, body mass index, presenting blood pressure, chronic hypertension status, antihypertensive treatment, anticoagulant or antiplatelet exposure, medication adherence, direct stress exposure, time from symptom onset to admission, transfer status, hematoma volume, radiographic location, functional outcome, and detailed cause-specific mortality. These variables were not consistently available in the linked spreadsheet-based administrative dataset used for the present analysis, which is why they could not be incorporated into the primary comparison. Fourth, although in-hospital mortality among strict spontaneous intracerebral hemorrhage patients was examined, the number of deaths was very small and did not permit meaningful inference regarding mortality differences between periods. Fifth, wartime changes in transport behavior, hospital diversion, referral pathways, neurosurgical triage, and protected-space reorganization may have influenced case capture. Sixth, although sensitivity analyses against March 2025 alone and a rolling 3-year March baseline showed a consistent direction of effect, these analyses were underpowered and did not reach statistical significance. Seventh, although comparison of March 2026 with prior March cohorts partially addresses seasonality by matching the calendar month, the current analysis cannot fully exclude seasonal or secular effects without all-month data and formal seasonal modeling. Finally, diagnosis-text phenotyping remains vulnerable to documentation variability despite the use of strict inclusion and exclusion criteria.

## 6. Conclusions

Within a preliminary single-center neurology/neurosurgery services dataset, March 2026 was temporally associated with a higher proportional burden of strict spontaneous intracerebral hemorrhage admissions compared with March cohorts from the preceding decade. This increase was not paralleled by acute ischemic stroke and was only weakly reflected in non-traumatic subarachnoid hemorrhage. Sensitivity analyses using March 2025 alone and a rolling 3-year March baseline were directionally consistent but underpowered and did not independently confirm statistical robustness. The findings should therefore be interpreted as a hypothesis-generating temporal association rather than evidence of causality or population-level incidence. Future multicenter, hospital-wide, and registry-based studies incorporating blood pressure, medication exposure, body mass index, mortality, transfer patterns, and all-month seasonal modeling are needed to determine whether this observation reflects a true increase in intracerebral hemorrhage occurrence, altered wartime case capture, or both.

## Figures and Tables

**Table 1:** Baseline characteristics of patients with strict spontaneous ICH: March 2026 versus pooled March 2016–2025.

Variable	March 2026 (n = 9)	March 2016–2025 (n = 59)	*p* Value
Age, mean ± SD, years	72.3 ± 4.1	65.8 ± 14.2	0.215
Age, median (IQR), years	71.2 (70.7–75.7)	68.2 (55.7–76.0)	0.215
Age ≥ 70 years, n (%)	7 (77.8)	25 (42.4)	0.073
Male sex, n (%)	6 (66.7)	43 (72.9)	0.702
Female sex, n (%)	3 (33.3)	16 (27.1)	0.702
Parenchymal cerebral ICH, n (%)	6 (66.7)	37 (62.7)	—
ICH with intraventricular extension, n (%)	2 (22.2)	4 (6.8)	0.177
Unspecified/generic intracranial hemorrhage, n (%)	1 (11.1)	15 (25.4)	—
Cerebellar ICH, n (%)	0	2 (3.4)	—
Brainstem/pontine ICH, n (%)	0	1 (1.7)	—

**Table 2:** Comparator cerebrovascular phenotypes in March 2026 versus pooled March 2016–2025.

Phenotype	March 2026	March 2016–2025	RR (95% CI)	*p* Value
Strict spontaneous ICH	9/223 (4.0%)	59/3632 (1.6%)	2.48 (1.25–4.94)	0.015
Acute ischemic stroke	17/223 (7.6%)	342/3632 (9.4%)	0.81 (0.51–1.29)	0.475
Non-traumatic SAH	6/223 (2.7%)	52/3632 (1.4%)	1.88 (0.82–4.33)	0.147

## Data Availability

The data presented in this study are available from the corresponding author upon reasonable request. Restrictions apply due to patient privacy, ethical considerations, and institutional policies, as the full dataset is derived from hospital-wide March admissions collected over approximately a decade and includes sensitive patient-level clinical information.
